# Designing Reliable Care Through Clinical Pathways: A Human Factors Framework

**DOI:** 10.3390/healthcare14152401

**Published:** 2026-08-05

**Authors:** Diego R. Hijano, Daniel A. Clark

**Affiliations:** 1 Department of Infectious Diseases, St. Jude Children’s Research Hospital, Memphis, TN 38105, USA; 2 Department of Pediatrics, University of Tennessee Health Science Center, Memphis, TN 38163, USA; 3 West Cancer Center & Research Institute, Memphis, TN 38138, USA; daclark1@memphis.edu; 4 School of Public Health, University of Memphis, Memphis, TN 38152, USA

**Keywords:** clinical pathways, human factors, healthcare quality, reliability, process improvement

## Abstract

**Highlights:**

**What are the main findings?**

- Clinical pathways can support healthcare reliability when designed as sociotechnical interventions informed by human factors engineering and systems principles rather than solely as standardization tools.
- Integrating human factors, workflow design, cognitive support, and organizational learning into pathway development provides a framework for safer, more adaptive, and more reliable care delivery.

**What are the implications of the main findings?**

- Healthcare organizations should approach clinical pathways as dynamic operational systems that support clinician performance, reduce variability, and strengthen patient safety across complex care environments.
- Applying human factors principles to pathway design may improve implementation success, clinician engagement, and sustainability of quality improvement initiatives in modern healthcare systems.

**Abstract:**

**Background/Objectives:** Healthcare delivery systems are inherently complex, and efforts to improve quality and safety often fail to achieve sustained, system-wide impact because of misalignment between work system design, care processes, and clinical practice. This article presents a practical, theory-informed framework that integrates human factors and systems thinking to guide the design and implementation of clinical pathways for reliable care delivery and system-level transformation. **Methods:** A conceptual framework was developed through synthesis of literature from human factors engineering, sociotechnical systems theory, and healthcare quality improvement. The framework organizes healthcare delivery into three interconnected domains—the work system, care processes, and outcomes—while positioning clinical pathways as the operational mechanism linking system design to care execution. Pediatric immunization was used as an illustrative case example. **Results:** The framework illustrates how misalignment in system design may contribute to variability in care processes and outcomes. Clinical pathways integrated into routine workflows and supported by human factors strategies—including decision-support heuristics, workflow simulation, and structured debriefing—may promote more reliable care delivery. Sustained implementation across teams and clinical settings is expected to reduce missed opportunities, improve consistency of care, and support progression toward system-level transformation. **Conclusions:** Clinical pathways can serve as practical tools for translating system design principles into reliable healthcare delivery when implemented using human factors and systems-based approaches. This framework provides healthcare leaders and quality improvement teams with a structured approach for designing, implementing, and scaling pathways to support safer, more reliable, and more consistent care delivery.

## 1. Introduction

Healthcare delivery systems are complex sociotechnical environments in which people, processes, technologies, and organizational structures interact to shape care delivery. Human factors science emphasizes that safe, effective, and patient-centered care emerges from work systems designed to align with human capabilities and limitations rather than relying on individual vigilance alone [1,2].

The Systems Engineering Initiative for Patient Safety (SEIPS) 2.0 framework provides a structured approach to understanding this complexity. In this model, a sociotechnical work system composed of persons, tasks, tools and technologies, organizational conditions, and environments gives rise to care processes that shape patient, professional, and organizational outcomes [1]. Outcomes therefore emerge from interactions among system components rather than from isolated actions.

This systems perspective also applies to organizational culture. Culture is shaped by the structures, workflows, and incentives that govern daily work. As a result, efforts to improve culture without redesigning underlying systems frequently fail to produce sustained change [3]. Reliability similarly emerges from systems that support consistent performance under varying clinical conditions and is best understood as a property of the system rather than of individual clinicians [1,4].

Clinical pathways represent a practical mechanism for translating system design into consistent care delivery, particularly in fragmented or time-constrained settings. By operationalizing evidence-based practices into structured workflows, pathways support point-of-care decision-making and reduce reliance on memory alone [5]. Their effectiveness, however, depends on integration into routine workflows. Poorly designed decision support, such as interruptive alerts, can increase cognitive load and disrupt workflow, limiting impact [6,7]. In contrast, pathways aligned with human factors principles support more reliable execution of care [2].

Evidence demonstrates that clinical pathways improve process adherence, reduce complications and length of stay, and, in some settings, decrease healthcare costs [8,9]. Integration of a revised pathway into the electronic health record for pediatric community-acquired pneumonia, for example, increased the use of first-line antibiotic therapy and shortened treatment durations across emergency department and urgent care settings [10].

Taken together, these frameworks suggest that improving healthcare reliability requires more than disseminating evidence or creating protocols. Healthcare organizations need practical mechanisms that translate system design principles into routine clinical workflows. Although existing frameworks such as SEIPS provide important insights into relationships among the work system, care processes, and outcomes, they do not explicitly describe how organizations can operationalize these concepts through pathway-based care delivery. This paper addresses that gap by presenting a human factors- and systems-based framework for clinical pathways, positioning pathways as operational tools for aligning the work system, care processes, and outcomes in support of reliable care delivery and system-level transformation.

## 2. Materials and Methods

This paper presents a conceptual framework developed through synthesis of literature and theoretical models in human factors engineering, sociotechnical systems theory, and healthcare quality improvement. The framework builds on the Systems Engineering Initiative for Patient Safety (SEIPS) 2.0 model as an organizing structure to examine relationships between the work system, care processes, and outcomes in healthcare delivery [1].

A targeted narrative review approach was selected because the objective of this work was conceptual integration and theory development rather than comprehensive evidence synthesis. Foundational and contemporary literature from human factors engineering, sociotechnical systems theory, clinical pathways, high reliability organizations, and healthcare quality improvement was identified through review of seminal frameworks, systematic reviews, and influential empirical studies. Priority was given to sources that examined relationships between work system design, care processes, implementation, and outcomes. Concepts recurring across these bodies of literature were iteratively mapped to the SEIPS 2.0 framework and synthesized into the proposed framework. Through this process, the work system, care processes, and outcomes were identified as the primary domains of healthcare delivery, while clinical pathways emerged as a practical operational mechanism for translating system design into routine clinical practice.

The framework organizes healthcare delivery into 3 interconnected domains: the work system (blunt end), care processes (activity), and outcomes (sharp end). Clinical pathways are positioned as the central mechanism for operationalizing system design within clinical workflows. Human factors principles, including cognition, workflow integration, and usability, inform pathway design and implementation within complex clinical environments.

Pediatric catch-up immunization in outpatient settings was selected as an illustrative case because it requires coordinated decision-making across clinicians, workflows, decision-support tools, and organizational processes; occurs within time-constrained encounters; and requires application of evidence-based guidelines. The case was mapped to the framework by identifying representative work system factors, care process activities, implementation strategies, and outcome measures relevant to immunization delivery. This mapping demonstrates how the framework can be operationalized in a real-world clinical setting to support consistent identification and delivery of recommended care across teams and clinical encounters.

This work does not represent a formal systematic review or empirical study, but rather a theory-informed framework intended to guide the design, implementation, and scaling of clinical pathways within healthcare systems. Institutional Review Board approval was not required because this work represents a conceptual framework and literature-based analysis and did not involve human participants, identifiable data, or patient-level information.

## 3. Results

### 3.1. From the Work System to Outcomes

Reliable healthcare delivery emerges from the interaction between the work system, care processes, and outcomes rather than from isolated interventions or individual performance alone.

Within this framework, clinical pathways serve as a mechanism for aligning system design with day-to-day care delivery and supporting more consistent execution of care. Transformation is therefore understood not as the result of a single intervention, but as the cumulative effect of sustained pathway-based processes applied across teams, settings, and clinical encounters over time.

**Figure 1:**
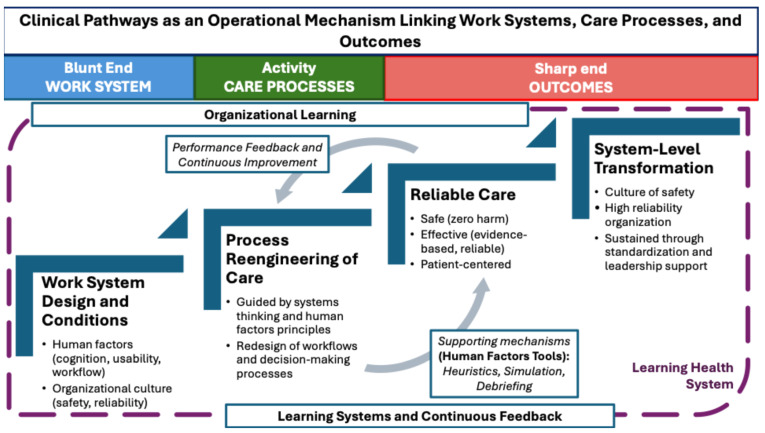
Conceptual framework linking clinical pathways to system-level transformation in healthcare.

Figure 1 illustrates the relationships among the work system, care processes, and outcomes within the proposed framework. Table 1 applies the framework to pediatric catch-up immunization, demonstrating how these domains interact within a real-world clinical setting.

Figure 1 presents a systems-based framework for improving quality and safety by linking work system design (blunt end), care processes (activity), and outcomes (sharp end), consistent with human factors frameworks such as SEIPS. At the blunt end, the work system is shaped by human factors (e.g., cognition, usability, workflow design) and organizational conditions, which influence variability and missed opportunities in care delivery. Within the activity layer, care processes translate system design into clinical practice through process reengineering, guided by systems thinking and human factors principles and supported by strategies such as heuristics, simulation, and structured debriefing. Clinical pathways serve as the central operational mechanism, embedding evidence-based guidance within routine workflows and enabling consistent execution of care across clinical encounters and teams. At the sharp end, these processes contribute to improved outcomes, including safe, effective, and patient-centered care. The framework is embedded within a learning health system characterized by organizational learning, continuous feedback, performance monitoring, and ongoing improvement. When sustained over time, these feedback mechanisms support adaptation of the work system and care processes, fostering continuous learning and system-level transformation characterized by increased reliability, standardization, and adaptability.

Table 1 illustrates the application of the conceptual framework across the work system (blunt end), care processes (activity), and outcomes (sharp end) using catch-up immunization in outpatient pediatrics. It maps key system elements, process steps, and expected outcomes, demonstrating how clinical pathways operationalize system design into consistent care delivery and support improved reliability.

### 3.2. Work System (Blunt End): System Design and Conditions

Within sociotechnical models of healthcare, the blunt end represents the work system: the structural and environmental conditions under which care is delivered. The SEIPS 2.0 framework defines the work system as interacting components including persons, tasks, tools and technologies, organizational structures, and the physical environment, all of which shape care processes and outcomes [1,2]. Risk and inconsistency emerge not from individual elements alone, but from how these components interact in practice.

Human factors are central to understanding and designing the work system. Clinicians operate under conditions of cognitive load, time pressure, and competing demands while navigating complex workflows and fragmented information systems. Poorly designed technologies and workflows can increase cognitive burden, contribute to errors, and negatively affect patient safety and clinician well-being [2,6]. In contrast, systems designed with attention to usability, workflow integration, and cognitive support facilitate decision-making and promote more consistent care delivery [2,11].

Organizational culture is also embedded within the work system and influences how care is prioritized and delivered. High reliability organizations demonstrate that sustained safety and performance depend on system characteristics such as sensitivity to operations, deference to expertise, and continuous attention to identifying and mitigating failure [12,13]. When reliability is not supported through standardized workflows, clear expectations, and accessible decision support, clinicians must rely on their judgment and memory, increasing the likelihood of inconsistent care and missed opportunities.

These dynamics are particularly evident in outpatient pediatric care, where preventive services such as immunizations compete with acute concerns, time constraints, and administrative demands. Missed opportunities for vaccination may arise from fragmented documentation, unclear workflows, competing priorities, and difficulty interpreting complex catch-up schedules [6,14]. In this context, inconsistency reflects differences in how care is delivered across similar situations, whereas reliability refers to the system’s ability to produce consistent outcomes despite operational complexity.

Understanding the blunt end as a system of interacting human, technological, and organizational elements highlights a central principle: inconsistency in care often reflects system design rather than individual performance [4]. Addressing this requires interventions that redesign the work system itself, creating conditions for more reliable and scalable care delivery.

These work system challenges correspond directly to the problems outlined in the Introduction, including fragmented information, competing priorities, workflow inefficiencies, and reliance on individual memory. Clinical pathways provide one mechanism for addressing these challenges by translating system design into structured care processes.

### 3.3. Care Processes (Activity): Process Reengineering and Clinical Pathways

At the center of the framework, the activity layer represents care processes, where system design is translated into clinical practice. While the work system defines the conditions under which care is delivered, care processes determine how those conditions are operationalized and experienced by clinicians and patients. Differences in outcomes are therefore shaped by how consistently care processes are executed in real-world settings [1,2].

Clinical pathways serve as a key mechanism for structuring these processes. Broadly defined, pathways are multidisciplinary plans of care that translate evidence-based guidelines into local workflows and actionable steps [11]. Evidence demonstrates that pathways improve adherence to recommended practices, reduce complications and length of stay, and, in some settings, decrease healthcare costs [5,8,9]. These findings support the role of pathways as tools for improving process performance in complex or time-constrained settings.

Within sociotechnical systems, pathways function not only as standardization tools but also as operational mechanisms that translate system design into consistent execution of care. By structuring decision-making, clarifying roles, and embedding key steps within workflows, pathways reduce reliance on individual memory and support more predictable performance across clinical encounters.

The effectiveness of pathways, however, depends on implementation within the clinical environment. When embedded within routine workflows and aligned with human factors principles, pathways can support decision-making, reduce cognitive burden, and facilitate coordination across care teams [2]. Human factors approaches provide practical methods to support this alignment, including heuristics and decision supports, workflow simulation, and structured debriefing to support continuous learning and adaptation. In contrast, pathways delivered through interruptive alerts or processes outside routine workflows are more likely to be bypassed or inconsistently applied [6,7]. Examples of these implementation strategies are outlined in Table 2.

Table 2 outlines practical human factors strategies that support the integration of clinical pathways into routine clinical workflows. For each strategy, it provides a description, an example in pediatric immunization, and practical applications to enhance decision-making, reduce cognitive burden, improve workflow integration, and support continuous learning.

In outpatient pediatric immunization, these principles are particularly relevant. Catch-up immunization requires coordinated clinical work involving multiple team members, tools, and processes, as detailed in Table S1. Accurate assessment of immunization status, interpretation of complex schedules, and execution within time-limited encounters create conditions in which missed opportunities are common. Clinical pathways can streamline this process by embedding immunization algorithms into workflows, integrating point-of-care decision support, and standardizing key steps in vaccine assessment and delivery [7,14]. Human factors-informed strategies such as simplified heuristics, workflow simulation, and structured team debriefings further strengthen pathway implementation and reliability. When applied consistently across teams and clinical encounters, these approaches reduce reliance on individual judgment and support more reliable delivery of recommended vaccines. In practice, successful implementation would be expected to increase vaccination rates, reduce missed opportunities for immunization, improve adherence to recommended schedules, and enhance workflow efficiency through earlier identification of vaccine needs and more standardized care processes. These outcomes are consistent with published evidence demonstrating that pathway-based approaches can improve adherence to recommended care and reduce variation in clinical practice [5,8,9,10].

Together, these elements illustrate how process reengineering, guided by human factors and operationalized through clinical pathways, transforms care processes from variable and clinician-dependent to more structured and reliable [1,2]. This transformation serves as the critical bridge between system design and outcomes, supporting high reliability and sustained improvement in care delivery [12,13].

### 3.4. Outcomes (Sharp End): Reliability and System-Level Transformation

At the sharp end of the framework, outcomes represent the observable results of care delivery, including patient outcomes, care quality, and system performance. Within sociotechnical models such as SEIPS, outcomes emerge from interactions between the work system and care processes rather than from the actions of individual clinicians alone [1,2]. Improvements in outcomes are therefore best understood as the downstream effect of well-designed systems and reliably executed care processes.

Reliability in healthcare refers to the consistent delivery of safe, effective, and patient-centered care across varying conditions and over time. High reliability organizations demonstrate that achieving such performance depends on system characteristics that support anticipation of failure, responsiveness to changing conditions, and continuous learning [12,13]. These organizations maintain sensitivity to frontline operations, defer to expertise, and continuously identify and mitigate potential failures before harm occurs.

Reliability is not achieved through episodic improvements but through sustained alignment between system design and care processes across teams and clinical encounters. As clinical pathways are implemented and integrated into routine practice, they reduce unwarranted variation and support more consistent execution of care. Over time, the cumulative effect of these changes shifts care delivery from reactive and variable to more disciplined and predictable systems of care.

In this context, transformation reflects the longitudinal result of sustained improvements in reliability. Beyond improved clinical outcomes, transformation includes the development of systems that support continuous learning, standardization, and adaptability in the face of complexity [4]. As reliability becomes embedded within care processes, organizations move beyond episodic quality improvement efforts toward integrated, system-based approaches to quality management [15,16]. In these settings, quality becomes an inherent property of care delivery rather than a separate organizational function [16]. Feedback loops, standardized processes, and leadership engagement work together to sustain performance and support ongoing adaptation.

Implementation of this framework in real-world settings, however, remains complex. Healthcare systems operate under constraints including time pressure, competing priorities, resource limitations, and variability in organizational culture, all of which can hinder the adoption and sustainability of clinical pathways. Resistance to change, often reflecting misalignment between pathway design, clinician workflows, and professional autonomy, may further reduce pathway effectiveness [6,17]. In addition, efforts to standardize care must be balanced against the need for clinical judgment, workflow efficiency, and organizational adaptability, reflecting inherent tradeoffs in complex sociotechnical systems [4]. For example, highly standardized vaccine assessment processes may improve reliability and reduce missed opportunities for care but can increase documentation burden and workflow complexity. Similarly, increasing pathway adherence may improve consistency while limiting flexibility in unusual clinical situations where individualized decision-making is appropriate. Organizations must continuously manage these efficiency-thoroughness tradeoffs through frontline engagement, performance monitoring, and iterative refinement of pathway design. Table S2 provides representative examples of these tradeoffs across the work system, care processes, and outcomes. Table 3 summarizes major implementation barriers and potential mitigation strategies.

Table 3 summarizes common barriers to the implementation and sustainability of clinical pathways across the domains of the conceptual framework. It highlights challenges related to human factors alignment, organizational resistance, workflow integration, cognitive burden, role clarity, adoption, measurement, and sustainability, along with representative mitigation strategies.

## 4. Discussion

Healthcare delivery systems are complex sociotechnical environments, and sustained quality improvement requires alignment between work system design, care processes, and clinical practice [1,2].

Building on the SEIPS 2.0 framework, this paper presents a conceptual framework linking the work system, care processes, and outcomes, positioning clinical pathways as a practical mechanism for translating system design into reliable care delivery [1]. The primary contribution of this framework is not the creation of a new sociotechnical theory but the integration of established human factors and systems concepts into an operational framework for pathway-based care delivery. While SEIPS 2.0 provides a valuable framework for understanding relationships among the work system, care processes, and outcomes, it does not explicitly identify how organizations can translate those relationships into routine clinical practice. The proposed framework extends this perspective by explicitly positioning clinical pathways as an operational mechanism that links system design to care delivery. By integrating pathway development, workflow design, implementation strategies, and continuous learning, the framework provides healthcare leaders and improvement teams with a practical approach for translating conceptual system design into reliable execution across clinical settings.

Clinical pathways were selected as the framework’s operational mechanism because they commonly serve as coordinating structures that integrate multiple implementation tools into a unified care process. In practice, pathways often incorporate standardized order sets, clinical decision support systems, checklists, care bundles, protocols, and workflow guidance to support consistent care delivery [7,11]. The intent of the framework is not to prioritize pathways over these alternative approaches, but rather to use pathways as an illustrative example of how healthcare organizations can operationalize system design. The underlying principles of the framework could also be applied through other implementation strategies that align the work system, care processes, and outcomes in support of reliable care delivery.

This perspective reframes pathways not as static protocols, but as tools for redesigning workflows, supporting decision-making, and enabling more consistent execution of care. Their effectiveness depends not only on the evidence they encode, but also on how well they are integrated into workflows and aligned with human capabilities [2,5]. Human factors strategies—including decision-support heuristics, workflow simulation, structured debriefing, and performance feedback—provide practical methods to support this alignment [2,18]. Several human factors principles are particularly important during pathway development and implementation. These include reducing unnecessary cognitive workload, designing workflows that fit existing clinical practice, standardizing routine tasks while preserving flexibility for clinical judgment, supporting team communication, and creating mechanisms for continuous feedback and learning. Operationalizing these principles may involve workflow mapping, simulation testing, usability assessment of decision-support tools, frontline stakeholder engagement, and iterative pathway refinement. Organizations can evaluate the extent to which pathways are human factors informed by assessing usability, workflow integration, clinician adoption, adherence patterns, user feedback, and the presence of unintended consequences such as workarounds or alert fatigue. These activities help ensure that pathways function as practical tools that support clinical work rather than additional requirements imposed on frontline teams.

The pediatric immunization example illustrates how gaps between policy and practice often emerge from system-level constraints rather than individual clinician performance [14]. In these settings, clinical pathways can reduce missed opportunities by embedding structured guidance into routine care processes [5,14]. Importantly, these improvements are not attributable to isolated interventions but to the cumulative effect of pathway-based processes applied across teams, clinical encounters, and care settings over time. This reinforces the principle that reliability is achieved through sustained system-level alignment rather than episodic improvement efforts [12,13].

Although pediatric immunization serves as the illustrative example throughout this manuscript, the framework is intended to support a broad range of healthcare improvement initiatives. Similar principles can be applied to sepsis recognition and treatment pathways, enhanced recovery after surgery programs, chronic disease management, medication safety initiatives, and transitions of care. Across these settings, reliable outcomes depend on alignment between work system design, care processes, implementation strategies, and frontline clinical practice. The framework is therefore intended for broad application in operationalizing evidence-based care across diverse clinical environments rather than being limited to immunization delivery.

At the outcome level, reliability emerges as a defining characteristic of high-performing systems. Consistent with principles of high reliability organizations, achieving reliable care requires standardized processes as well as systems that support continuous learning, responsiveness to frontline conditions, and alignment between leadership priorities and daily practice [12,13]. At the same time, implementation is shaped by real-world constraints, including competing priorities, workflow tradeoffs, resource limitations, and resistance to change [4,6]. These challenges are not peripheral but central to translating conceptual models into sustained practice. Organizations must therefore anticipate implementation barriers through frontline engagement, workflow redesign, and sustained leadership support.

Organizational culture also plays a reciprocal role within the proposed framework. While culture is influenced by work system design, leadership priorities, and care processes, it may also shape pathway adoption, clinician engagement, and long-term sustainability. Cultural characteristics such as psychological safety, trust, openness to learning, and leadership support can facilitate implementation by encouraging participation in pathway development, reporting of challenges, and continuous refinement of care processes. Conversely, successful pathway implementation and sustained improvements in reliability may reinforce a culture of continuous improvement, shared accountability, and organizational learning. Recognizing this bidirectional relationship highlights the importance of addressing both technical and cultural dimensions of system change [3,17].

Patient-centered considerations are also essential to pathway design and implementation. Although pathways aim to promote reliable delivery of evidence-based care, they should not replace individualized decision-making or patient preferences. Patients and families can contribute valuable perspectives regarding care experiences, barriers to adherence, workflow challenges, and outcome priorities. Incorporating patient and family input through co-design approaches may improve pathway usability, acceptability, and alignment with patient needs. In practice, pathways should support shared decision-making by providing structured guidance while preserving flexibility to accommodate individual clinical circumstances, patient values, and informed preferences [19].

Although reliability, consistency, and standardization are closely related, they represent distinct concepts within the framework. Standardization refers to the deliberate design of processes, workflows, tools, or procedures intended to reduce unnecessary variation in care delivery. Consistency reflects the degree to which those processes are executed similarly across clinicians, teams, settings, and encounters. Reliability represents the ability of the system to consistently achieve intended outcomes under varying conditions and over time. In this context, standardization serves as a mechanism for promoting consistency, while consistency contributes to reliability. However, reliability ultimately depends not only on standardized processes but also on adaptation, learning, and effective system design.

The proposed framework is complementary to established implementation science approaches. Frameworks such as the Consolidated Framework for Implementation Research (CFIR), Reach, Effectiveness, Adoption, Implementation, and Maintenance (RE-AIM), and Normalization Process Theory provide valuable methods for understanding factors that influence adoption, implementation, sustainability, and integration of interventions into routine practice. In contrast, the present framework focuses primarily on how work system design, care processes, and human factors principles interact to support reliable care [20,21,22]. Implementation science frameworks may therefore serve as useful companions during pathway deployment by helping organizations identify contextual barriers, evaluate implementation outcomes, and understand factors influencing long-term sustainability. Future work may benefit from integrating these perspectives to strengthen both pathway implementation and evaluation efforts.

Successful implementation of the framework requires a structured but adaptable approach. Organizations can begin by engaging frontline stakeholders, mapping existing workflows, identifying sources of variation or missed opportunities, and co-designing pathway-supported processes that align with local practice. Human factors methods such as workflow simulation, usability assessment, iterative testing, and structured feedback mechanisms can then be used to refine implementation. Integration within electronic health records, clear role definitions, performance dashboards, and ongoing team training may further support pathway adoption and sustainability. Because healthcare environments vary considerably in resources, staffing, technology infrastructure, and patient populations, pathway implementation should be tailored to the local context while preserving core evidence-based elements.

Although clinical pathways can support reliability and consistency, their implementation may also introduce unintended consequences if poorly designed or inadequately integrated into clinical workflows. Potential challenges include workflow disruption, excessive standardization, alert fatigue, reduced clinician engagement, and the development of workarounds that bypass intended processes. Human factors principles can help mitigate these risks by emphasizing usability, workflow compatibility, frontline involvement, and iterative refinement. Importantly, the framework does not assume that all patients should follow identical care trajectories. Rather, pathways are intended to support evidence-informed decision-making while preserving clinician judgment and adaptability when individual patient needs, preferences, or clinical circumstances warrant deviation from standardized recommendations. Reliable care therefore reflects the consistent application of sound clinical processes, not rigid adherence to protocols regardless of context.

This work should also be interpreted in light of its limitations. The proposed framework is theory-informed but has not yet been empirically validated. Although pediatric immunization is used as an illustrative example, the framework has not been prospectively tested across multiple healthcare settings or clinical conditions. In addition, variability in organizational culture, health information technology infrastructure, workforce resources, and leadership engagement may influence implementation and scalability. Future research should evaluate the application of this framework across diverse clinical environments and care processes to determine its effectiveness and generalizability, as well as its impact on reliability, safety outcomes, clinician experience, and operational performance. Potential implementation outcomes may include pathway adoption, acceptability, feasibility, fidelity, sustainability, and clinician engagement. Process measures could assess workflow integration, adherence to pathway components, reduction in missed opportunities for care, and consistency of care delivery across providers and settings. Reliability outcomes may include variation in care processes, achievement of evidence-based practices, and patient safety or quality indicators relevant to the clinical context. Appropriate study designs may include prospective quality improvement initiatives, mixed-methods implementation studies, multicenter evaluations, and comparative effectiveness analyses examining pathway-supported versus usual care delivery. Findings from such studies could inform future refinement of the framework and strengthen its practical application across healthcare systems.

This work presents a conceptual framework rather than a standardized intervention or implementation protocol. As such, it is not intended to prescribe a single reproducible model for all healthcare settings. Instead, it provides a theory-informed structure that organizations can adapt to their local context, resources, workflows, patient populations, and implementation priorities while preserving its underlying human factors and systems principles. This adaptability is intended to support implementation across diverse clinical environments while maintaining the framework’s conceptual integrity.

Together, these findings suggest that improving healthcare quality requires a shift from isolated improvement initiatives toward integrated, system-based approaches to care delivery [15,16]. Clinical pathways, when developed and implemented through a human factors-informed approach, offer a practical and scalable mechanism to support this transition. By aligning work system design with care processes and reinforcing consistent execution over time, pathway-based approaches provide a foundation for continuous learning in healthcare systems [1,2,12,23].

## 5. Conclusions

This paper presents a conceptual framework that integrates human factors and systems thinking to support reliable care delivery through clinical pathways. By linking work system design, care processes, and outcomes, the framework illustrates how pathways can operationalize evidence-based care within routine clinical workflows.

Clinical pathways should be understood not as static protocols, but as adaptable tools that support workflow integration, clinician judgment, patient-centered care, and continuous learning. When designed and implemented using human factors principles, pathway-based approaches may help healthcare organizations reduce unnecessary variation, strengthen reliability, and support sustained system-level improvement.

## Figures and Tables

**Table 1:** Translating the Conceptual Framework into Clinical Practice.

Framework Component	Key Elements	Application to Pediatric Immunization	Expected Impact
Work System (Blunt End)	Human factors, workflow design, EHR usability, organizational culture	Fragmented documentation, competing priorities, time constraints, variability in clinician knowledge and workflows	Missed opportunities, inconsistent identification of immunization needs, variability in care delivery
Care Processes (Activity)	Clinical pathways, decision support, workflow redesign, role clarity	Embedded immunization algorithms, EHR-integrated prompts, standardized assessment and ordering processes	Improved identification of eligible patients, streamlined care delivery, reduced cognitive burden
Outcomes (Sharp End)	Reliability, safety, effectiveness, patient-centered care	Increased vaccination rates, reduced missed opportunities, improved adherence to immunization schedules	More consistent delivery of recommended care, improved population health outcomes
System-Level Transformation	Standardization, learning systems, leadership support, feedback loops	Sustained pathway implementation, performance monitoring, integration into routine care practices	Development of a culture of reliability, continuous improvement, and scalable system-wide impact

**Table 2:** Human Factors Strategies to Support Clinical Pathway Implementation in Pediatric Immunization.

Human Factors Strategy	Description	Example in Pediatric Immunization	Practical Application and Expected Impact
Heuristics/Decision Supports	Simplified rules or prompts that guide decision-making at the point of care	EHR prompt: “Child overdue for DTaP—eligible today” with pre-selected orders	Embed default orders and visual cues in EHR to reduce cognitive load, avoid missed vaccines, and improve pathway adherence
Workflow Simulation	Testing and refining processes before or during implementation to identify bottlenecks and inefficiencies	Simulating a clinic visit to determine when immunization status is reviewed (rooming vs. physician visit)	Conduct rapid-cycle simulations with staff to optimize workflow efficiency and pathway integration
Structured Debriefing	Systematic reflection after clinical sessions to identify failures, gaps, and opportunities for improvement	End-of-day team review of missed vaccination opportunities	Implement brief team huddles or weekly reviews to identify patterns, support learning, and improve reliability
Role Standardization	Clarifying responsibilities across team members to reduce ambiguity and missed steps	Medical assistant reviews immunization registry; physician confirms and orders vaccines	Define and standardize team roles for immunization assessment and delivery to improve accountability, reduce missed handoffs, and support consistent pathway execution
Visual Management Tools	Use of visual cues to make information accessible and actionable	Immunization status displayed on EHR dashboard or room whiteboard	Use dashboards or color-coded indicators to highlight vaccine gaps during visits, improve situational awareness, and facilitate timely intervention
Feedback Loops/Performance Monitoring	Providing data to teams on performance to support continuous improvement	Clinic-level reports on missed opportunities for vaccination	Share regular performance data with teams and incorporate findings into quality improvement discussions to reinforce learning, monitor pathway adherence, and drive continuous improvement

Abbreviations: DTaP, diphtheria, tetanus, and acellular pertussis vaccine; EHR, electronic health record. Human factors strategies are intended to support pathway adoption, workflow integration, reduction of missed opportunities for care, and continuous improvement through feedback and learning.

**Table 3:** Implementation Barriers to Pathway-Based Care Across the Sociotechnical System.

Framework Domain	Barrier Type	Description	Example in Clinical Pathways	Example in Pediatric Immunization	Practical Implication/Mitigation Strategy
Work System (Blunt End)	Human factors misalignment	Tools, workflows, and interfaces are not aligned with clinician cognition or workflow	Complex EHR navigation, excessive clicks, poor visibility of pathway steps	Immunization history difficult to locate; eligibility not readily visible	Increased cognitive burden and missed opportunities despite available information; mitigate through user-centered design, workflow testing, and simplification of EHR interfaces
	Organizational resistance	Misalignment between pathway design, clinician workflows, and professional autonomy leads to active or passive resistance to standardized processes	Clinicians bypass pathways, selectively adopt components, or revert to prior practices	Preference to defer vaccines or rely on individual judgment rather than structured catch-up protocols	Resistance reflects system misalignment; mitigate through clinician engagement, co-design, and alignment of workflows and incentives
	Resource constraints	Limited time, staffing, or infrastructure to support pathway use	No protected time or staffing support for pathway integration	Short visits prioritize acute concerns over preventive care	Incomplete implementation and inconsistent execution; mitigate through leadership support, prioritization of high-value pathway components, and allocation of implementation resources
Care Processes (Activity)	Workflow fragmentation	Pathways are not embedded within actual care delivery processes	Pathway steps require separate documentation or occur outside routine workflow	Immunization status reviewed late or inconsistently during visit	Missed opportunities due to poor timing and integration; mitigate by embedding pathway activities into routine clinical workflows and existing documentation processes
	Cognitive overload	Excessive prompts, alerts, or complex decision steps overwhelm clinicians	Alert fatigue from multiple decision support tools	Vaccine prompts ignored or overridden	Reduced effectiveness of decision support and emergence of workarounds; mitigate by minimizing unnecessary alerts and optimizing decision-support usability
	Role ambiguity	Lack of clarity regarding responsibilities across team members	Unclear ownership of pathway steps	Confusion between medical assistant and physician on vaccine assessment and ordering	Gaps in execution, duplication, or omission of tasks; mitigate through clear role definition, team training, and standardized responsibilities
Outcomes/System-Level	Inconsistent adoption	Uneven uptake across teams, clinics, or settings	Some units fully implement pathways while others do not	Variability in vaccination practices across providers or clinics	Persistent variation in care delivery; mitigate through standardized implementation approaches, leadership oversight, and performance monitoring
	Measurement and feedback gaps	Lack of data systems to monitor performance and outcomes	No tracking of pathway adherence or missed opportunities	Limited visibility of missed vaccination rates	Inability to identify gaps or drive improvement; mitigate through development of performance dashboards, feedback loops, and regular review of outcomes
	Sustainability challenges	Difficulty maintaining changes over time after initial implementation	Decline in adherence once initial focus diminishes	Reduced consistency in vaccine delivery over time	Erosion of gains and limited long-term impact; mitigate through continuous monitoring, reinforcement, leadership commitment, and integration into routine operations

## Data Availability

No new data were created or analyzed in this study. Data sharing is not applicable to this article.

## Supplementary Materials

The following supporting information can be downloaded at: https://www.mdpi.com/article/10.3390/healthcare14152401/s1, Table S1: Example of Collaborative Clinical Work: Catch-Up Immunization in Outpatient Pediatrics; Table S2: Tradeoffs in Scaling Clinical Pathways for Reliable Care Delivery.

## Abbreviations

The following abbreviations are used in this manuscript:

CFIR	Consolidated Framework for Implementation Research
DTaP	Diphtheria, tetanus, and acellular pertussis vaccine
EHR	Electronic health record
RE-AIM	Reach, Effectiveness, Adoption, Implementation, and Maintenance
SEIPS	Systems Engineering Initiative for Patient Safety
