# Supplementary material for: Designing Reliable Care Through Clinical Pathways: A Human Factors Framework
Source: Healthcare (Basel). 2026 Aug 5;14(15):2401. doi: 10.3390/healthcare14152401 (PMC13464977; doi:10.3390/healthcare14152401)
Supplement: Supplementary file 1 [file healthcare-14-02401-s001.zip › healthcare-4371448-supplementary.pdf]

Supplementary Table S1. Example of Collaborative Clinical Work: Catch-Up Immunization in Outpatient Pediatrics.

**Collaborative work:** Work in which healthcare professionals and patients (and/or caregivers) are jointly involved in care delivery and decision-making.<br>**Example:** Catch-up immunization during an outpatient pediatric visit.
**Agent(s):** Pediatrician, nurse, medical assistant, patient (child), parent/caregiver<br>**Co-agent(s):** Front desk staff, immunization registry systems, pharmacists, public health systems
**Work system factors:**<br>• **Person(s):** Clinician knowledge of immunization schedules, experience with catch-up protocols, caregiver beliefs, vaccine hesitancy, and health literacy<br>• **Task:** Complexity of catch-up immunization schedules, competing priorities during visits (acute concerns vs preventive care), and time constraints<br>• **Tools/technology:** Usability of the electronic health record (EHR), integration with immunization registries, availability of clinical decision support, and vaccine inventory systems<br>• **Organization:** Clinic workflow design, staffing levels, role clarity among team members, prioritization of preventive care, and performance measurement systems<br>• **Internal environment:** Clinic layout, room turnover efficiency, time pressure, and competing documentation demands<br>• **External environment:** State immunization policies, school requirements, vaccine availability, and public trust in vaccines<br>These factors interact dynamically to shape the performance of immunization delivery.
**Process:**<br>The process of delivering catch-up immunizations includes identifying immunization status, interpreting vaccine schedules, determining vaccine eligibility, engaging caregivers in shared decision-making, ordering and administering vaccines, documenting administration, and scheduling follow-up doses. Performance at each step can be influenced by different configurations of work system factors. Clinical pathways can support this process by embedding decision algorithms into workflows, integrating decision support within the EHR, and clarifying roles across the care team.
**Outcomes:**<br>• **Proximal outcomes:** Accurate identification of immunization gaps, timely administration of recommended vaccines, reduced missed opportunities, and clinician workload and cognitive burden

- **Distal outcomes:** Improved vaccination coverage rates, reduced incidence of vaccine-preventable diseases, improved patient and caregiver trust, reduced variability across providers, and enhanced system-level reliability

Supplementary Table S2. Tradeoffs in Scaling Clinical Pathways for Reliable Care Delivery.

Domain	Tradeoff	Description	Benefit	Risk	Example in Pediatric Immunization	Strategic Consideration
**Clinical Decision-Making**	Standardization vs Flexibility	Structured pathways versus individualized clinical judgment	Greater consistency and reduced variation	Reduced adaptability in complex or atypical cases	Deviation from catch-up schedule for medically complex patients	Define clear boundaries where clinical discretion is appropriate within pathways
	Automation vs Clinician Control	EHR-driven prompts versus clinician-led decisions	Reduced cognitive burden; improved adherence to recommendations	Over-reliance on automation or inappropriate overrides	Accepting or ignoring vaccine prompts without full assessment	Design decision support to augment—not replace—clinical reasoning
**Care Delivery & Workflow**	Efficiency vs Thoroughness (ETTO)	Faster workflows versus comprehensive evaluation	Increased throughput; reduced time burden	Missed steps or incomplete assessment	Skipping full immunization review due to time pressure	Protect critical safety steps while streamlining workflow (Hollnagel, 2009)
	Simplicity vs Completeness	Simplified guidance versus	Easier adoption and usability	Oversimplification may omit important nuances	Simplified vaccine	Use layered pathway design (simple front-

		comprehensive detail			schedules may miss edge cases	end with access to detailed guidance)
**System & Organizational Performance**	Scalability vs Local Adaptation	Standardized pathways across settings versus local customization	Enables system-wide consistency and spread	Poor fit with local workflows or team structures	Workflow mismatch between primary care and subspecialty clinics	Standardize core elements while allowing local adaptation in implementation
	Short-term Investment vs Long-term Reliability	Upfront effort and resources versus sustained performance gains	Long-term improvement in outcomes, efficiency, and reliability	Initial disruption, resource burden, slower early adoption	Time and effort required to redesign immunization workflows and train teams	Frame pathway implementation as a long-term strategic investment rather than a short-term intervention
